# Nanoparticles as Delivery Systems for Antigenic Saccharides: From Conjugation Chemistry to Vaccine Design

**DOI:** 10.3390/vaccines12111290

**Published:** 2024-11-19

**Authors:** Marie-Jeanne Archambault, Laetitia Mwadi Tshibwabwa, Mélanie Côté-Cyr, Serge Moffet, Tze Chieh Shiao, Steve Bourgault

**Affiliations:** 1 Department of Chemistry, Université du Québec à Montréal, C.P.8888, Succursale Centre-Ville, Montreal, QC H3C 3P8, Canadamwadi_tshibwabwa.laetitia@courrier.uqam.ca (L.M.T.);; 2 Quebec Network for Research on Protein Function, Engineering and Applications (PROTEO), Montreal, QC H3C 3P8, Canada; 3 The Center of Excellence in Research on Orphan Diseases-Fondation Courtois (CERMO-FC), Montreal, QC H3C 3P8, Canada; 4 Glycovax Pharma Inc., Laval, QC H7V 5B7, Canada; smoffett@glycovax.com (S.M.); tcshiao@glycovax.com (T.C.S.)

**Keywords:** vaccines, polysaccharides, antigens, glycoconjugates, nanoparticles, nanocarriers, conjugation, immune responses, liposomes

## Abstract

Glycoconjugate vaccines have been effective in preventing numerous bacterial infectious diseases and have shown recent potential to treat cancers through active immunotherapy. Soluble polysaccharides elicit short-lasting immune responses and are usually covalently linked to immunogenic carrier proteins to enhance the antigen-specific immune response by stimulating T-cell-dependent mechanisms. Nonetheless, the conjugation of purified polysaccharides to carrier proteins complexifies vaccine production, and immunization with protein glycoconjugates can lead to the undesirable immunogenic interference of the carrier. Recently, the use of nanoparticles and nanoassemblies for the delivery of antigenic saccharides has gathered attention from the scientific community. Nanoparticles can be easily functionalized with a diversity of functionalities, including T-cell epitope, immunomodulator and synthetic saccharides, allowing for the modulation and polarization of the glycoantigen-specific immune response. Notably, the conjugation of glycan to nanoparticles protects the antigens from degradation and enhances their uptake by immune cells. Different types of nanoparticles, such as liposomes assembled from lipids, inorganic nanoparticles, virus-like particles and dendrimers, have been explored for glycovaccine design. The versatility of nanoparticles and their ability to induce robust immune responses make them attractive delivery platforms for antigenic saccharides. The present review aims at summarizing recent advancements in the use of nano-scaled systems for the delivery of synthetic glycoantigens. After briefly presenting the immunological mechanisms required to promote a robust immune response against antigenic saccharides, this review will offer an overview of the current trends in the nanoparticle-based delivery of glycoantigens.

## 1. Introduction

Vaccination constitutes one of the most effective strategies for preventing infectious diseases and has recently demonstrated the potential to treat cancers through active immunotherapy [1,2]. Vaccines mainly act by training the immune system to recognize and tackle pathogens, such as viruses and bacteria, and this was historically achieved by introducing a killed, or attenuated, pathogen [3]. The immune system of the immunized host responds by generating antibodies and immune cells that specifically recognize the pathogen upon subsequent exposures [4]. These conventional vaccines based on live-attenuated and inactivated pathogens have played key roles in preventing serious infectious diseases for decades [3,4]. More recently, novel approaches such as nucleic acid, viral vector and subunit vaccines have provided safer alternatives for inducing potent and long-lasting antigen-specific immune responses and improving vaccine safety [3,4]. Moreover, active immunotherapy, which aims at harnessing immune cells and immune memory to target cancer cells, has emerged as a promising strategy to fight cancers [1,5].

A promising approach for vaccine design and immunotherapy involves the use of polysaccharides as antigens for subunit vaccines. Polysaccharides are complex carbohydrates that can be found on the surface of all human cells and microbes, including bacteria and viruses [6]. Glycans are highly specific to certain pathogens and, to some extent, to cancer cells, making them potential antigens for vaccination. However, antigenic glycans usually elicit a short-lasting immune response that fails to generate T-cell-dependent memory B-cells [6,7]. Furthermore, polysaccharides induce very weak antigen-specific immune responses in young children and immunocompromised patients [6,8]. To overcome these limitations, saccharidic antigens are usually covalently linked to an immunogenic protein carrier, leading to robust and long-lasting a T-cell-dependent immune response, allowing processes such as class switching and affinity maturation to take place [4,6,8]. The first glycoconjugate vaccine, which was against *Haemophilus influenzae* type b, used the diphteria toxoid as an immunogenic protein carrier and was licensed in the late 1980s [2,7,9]. To this day, glycoconjugates used in clinics include vaccines against *Haemophilus influenzae*, *Neisseria meningitidis*, *Streptococcus pneumoniae* and *Salmonella typhi* [2,10]. Nonetheless, challenges associated with the production and manufacturing of glycoconjugate vaccines as well as their efficacy remain to be addressed. Glycoantigens are usually isolated from bacterial cultures, and this complex, multistep procedure results in low yields and, often, the high level of heterogenicity of the glycan mixtures. The conjugation of the isolated bacterial polysaccharides to the carrier protein is technically challenging because the glycoantigen needs to be chemically activated, which can lead to its degradation and/or the alteration of its structure by hydrolysis or oxidation, depending on the chemistry employed [8,11]. This leads to issues with batch-to-batch reproducibility and limits widespread use, particularly in areas with limited resources [8]. Furthermore, the undesirable interference of immunogenicity between the carrier protein and the saccharide unit(s) can result in antigen competition and carrier-induced epitope suppression (CIES) [10,12]. CIES occurs when pre-existing immunity against an immunostimulant carrier protein can lead to a diminished antibody response against the antigen conjugated to the same carrier [12].

Ongoing research in the field of glycoconjugate vaccines aims at addressing these limitations by developing novel strategies to enhance safety, efficacy, production and accessibility. With recent advancements in nanotechnology, there is huge potential for the development of nanoparticle-based delivery systems for glycoantigens that can overcome the challenges associated with protein-based glycoconjugates. Notably, nanoparticles can protect the saccharidic antigens from degradation and can enhance their uptake by immune cells, ultimately leading to a robust and prolonged immune response [8,13,14]. Furthermore, by precisely controlling their shape, size, surface chemistry as well as the degree of functionalization with immunological components, it is possible to finely modulate and polarize the immune response [14,15,16]. In this context, this short review aims at summarizing the recent advancements in the use of nano-scaled systems for the delivery of synthetic glycoantigens. After briefly presenting the immunological mechanisms necessary to promote a robust immune response against antigenic saccharides, we will present a broad overview of the current trends in the nanoparticle-based delivery of glycoantigens, exploring opportunities and challenges associated with this strategy. While several comprehensive reviews focusing on the design of glycoconjugate vaccines to fight cancers or bacterial infections have been published over the last few years [2,7,10,17,18,19,20,21], the present short review focuses exclusively on the preparation and use of (semi)-synthetic glycoconjugate nanoparticles as next-generation vaccines.

## 2. Immune Responses Against Antigenic Polysaccharides

**Figure 1:**
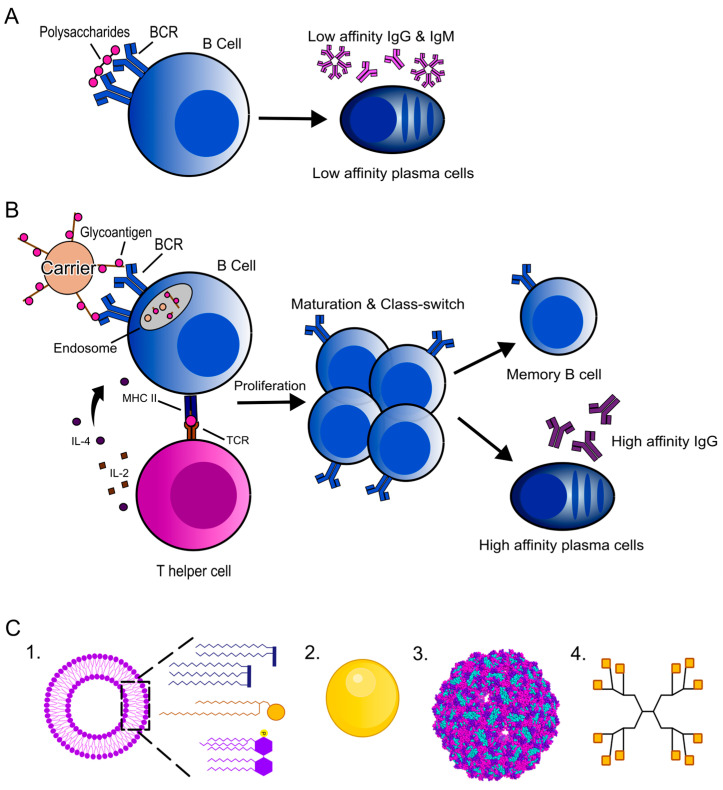
Schematic representation of the immune processing of (**A**) polysaccharides and (**B**) glycoconjugates leading to cytokine production and antibody secretion. Glycoconjugates can be processed intracellularly and are displayed as glycopeptides on B-cell via MHC II, allowing for recognition by T-cells. Co-stimulation between B- and T-cells leads to cytokine release and B-cell activation. This T-cell-dependent response generates high-affinity, class-switched antibodies and memory cells. BCR: B-cell receptor; MHC: Major histocompatibility complex; TCR: T-cell receptor; IL: Interleukin. (**C**) Schematic representation of glycoconjugate nanoparticles. (1) Liposomes that can incorporate immune activators such as Pam2, Pam3, alpha galactosylceramide, and MPLA. (2) Gold nanoparticles. (3) Virus-like particles, such as bacteriophage Q beta (PDB: 1QBE). (4) Dendrimers such as tetravalent lysine core dendrimer, represented here with N-acetylgalactosamine.

Carbohydrates are essential components for cellular functions and can be found on the cell surface of all living organisms, from bacteria to mammals [8]. Pathogenic bacteria, fungi and parasites expose polysaccharides on their surface that play pivotal roles in initiating infection, acting as virulent factors and/or aiding pathogens in evading the immune system of the infected hosts [6,7]. Cancer cells also display an overabundance of specific glycan structures on the outer leaflet of their plasma membrane, which can be used to distinguish tumor cells from healthy cells [17,22]. The administration of isolated and soluble polysaccharides results in a short-lived immune response associated with T-cell-independent immunity, which limits the presentation of antigen-presenting cells (APCs) on major histocompatibility complexes (MHCs) [7,8,22]. Thus, polysaccharides fail to activate T-cells, which is essential for inducing long-lasting immunity, resulting in the secretion of low-affinity IgM antibodies through T-cell-independent mechanisms [23] (Figure 1A).

To overcome this limitation, polysaccharides are usually covalently linked to immunogenic carrier proteins, such as tetanus toxoid or diphtheria toxoid. This conjugation enhances the immune response by stimulating T-cell-dependent mechanisms, which is possible because of the presence of T-cell epitopes within the protein sequence [8,23]. The stimulation of B-cells and subsequent production of high-affinity IgG antibodies requires antigen binding with B-cell receptors along with stimulation by cytokines secreted by T helper cells (Figure 1B) [8,22,23,24]. Portions of the glycoconjugate including carbohydrates bind with and induce the clustering of B-cell receptors. After internalization into a B-cell endosome, the glycoconjugate molecule undergoes processing, with proteases breaking it into smaller peptides and glycopeptides, resulting in the generation of glycan-peptides. Certain fragments can then be loaded onto MHC II molecules, leading to the cell surface presentation of peptides and/or glycopeptides to the CD4 T-cells to trigger an adaptive response. In both cases, this leads to the recognition of the peptide and/or the glycan by the T helper cell, which in turn stimulates the secretion of cytokines, including IL-4 and IL-2 [23]. This induces the maturation of associated memory B-cells, prompting these cells to produce high-affinity carbohydrate-specific IgG antibodies [8,23,24,25]. The mechanisms by which carbohydrate-specific antibodies contribute to protection against the initial infection, the growth and/or the propagation of microbial pathogens involve several key processes. For instance, the activation of the complement system helps to identify target pathogens, or cancer cells, for their elimination by the host organism [26]. Moreover, the opsonization of the pathogens facilitates their phagocytosis and destruction by immune cells [26]. Moreover, the binding of antibodies to cancer cells’ glycoproteins may also interfere with critical survival signaling, such as that of growth factors, for attachment to the extracellular matrix or surrounding cells, or for nutrient uptake. Together, these mechanisms allow carbohydrate antibodies to play an important role in the immune response against a variety of infectious agents, as well as towards cancer cells. For detailed information regarding the induction of immune response against polysaccharides and the subsequent protection conferred, we encourage readers to consult the comprehensive review of Kappler and colleagues [27].

## 3. Nanocarriers for Saccharide Antigens

The use of carrier proteins in glycoconjugate vaccines was a breakthrough as it addressed the poor immunogenicity of isolated polysaccharides. Carrier proteins were found to enhance the recognition of saccharidic antigens by the immune system, leading to the activation of T-cells and the subsequent long-lasting production of antibodies by B-cells, thus significantly improving the efficacy of glycovaccines [8,13,22,23,24,25]. Five carrier proteins are currently licensed for human glycoconjugate vaccines: diphtheria toxoid (DT), tetanus toxoid (TT), cross reacting material 197 (CRM197), *Haemophilus* protein D (PD) and the outer membrane protein complex of serogroup B meningococcus (OMPC) [6,10,21]. In contrast, there are only four types of bacteria for which a corresponding glycoconjugate vaccine has been approved for human use: *Haemophilus influenzae*, *Neisseria meningitidis*, *Streptococcus pneumoniae* and *Salmonella typhi* [6,10,21]. Immunization with nanoparticle-based delivery systems presents numerous benefits in comparison to conventional protein conjugate vaccines. Through rational engineering, nanoparticles can mimic pathogens and present a repetitive display of antigens on their surface [28,29]. Additionally, their nanoscale size, in the range of most pathogens, enables efficient adsorption by APCs, enhancing the presentation of antigens to adaptive immune cells, and facilitates their passive diffusion into lymph nodes (LNs) [30]. Furthermore, the shape of nanoparticles can also influence the immune response. Studies have shown that rod-shaped structures tend to be more pro-inflammatory compared to spherical particles [31,32]. Conjugation to nanoparticles also leads to improved stability and the prolonged release of antigens [28,29]. Nanoplatforms used for the presentation and delivery of glycoantigens can be classified into various types based on their chemical nature and supramolecular structure, including lipid vesicles, metallic nanoparticles, virus-like particles (VLPs) and dendrimers.

### 3.1. Liposomal Nanocarriers

Liposomal carriers, which are usually assembled from amphiphilic molecules, can present clusters of carbohydrate antigens on their surface, which help to stimulate B-cells by presenting a repetitive structure to cross-link with the B-cell receptors [27]. These carriers often have intrinsic self-adjuvanting effects by activating different immune pathways, including those involving Toll-like receptors (TLRs) and natural killer T (NKT) cells [33]. For instance, the adjuvant AS01 used in human vaccines is constituted of liposomes prepared from two immunostimulant lipids: 3-O-desacyl-4ʹ-monophosphoryl lipid A (MPLA) and the saponin QS-21 [34,35]. Liposomes are usually spherical nanovesicles composed of a lipid bilayer that can be conjugated with and/or encapsulate antigens [33,34]. Their size dictates which immune response will be activated. For instance, liposomes approximatly 100 nm in diameter will promote Th2-balanced responses, while larger ones, with a diameter of 400 nm or greater, will promote Th1-balanced reponses [33]. These nanoparticles have been extensively studied for antigen delivery due to their biocompatibility and ability to carry a diversity of antigens, as well as immunomodulating molecules (Table 1) [29,33]. Liposomes enhance the stability of antigens by protecting them against physiologycal processes, including enzymatic degradation, and promoting their uptake by APCs, which is often associated with the similar phospholipid composition between the plasma membrane of APCs and liposomes, [36,37]. Altogether, this leads to improved antigen-specific immune responses.

The Thomsen nouveau (Tn) antigen, i.e., N-acetylgalactosamine (GalNAc), which is frequently highly abundant on the surface of cancer cells, was conjugated to cholesterol and incorporated in liposomes of approximately 150 nm that encapsulated the TLR9 agonist unmethylated 5′-C-phosphate-G-3′ (CpG) (Table 1) [49]. This glycoliposome showed a strong interaction with an anti-Tn-specific antibody and improved the presentation of the Tn antigen by both bone-marrow-derived dendritic cells (BMDCs) and spleen-derived B-cells, which are essential for eliciting robust T- and B-cell-mediated immune responses [38]. The evaluation of immunogenicity in mice revealed that this liposomal glycovaccine stimulates the robust production of anti-Tn-specific IgG, as well as the secretion of IFN-γ [38].

Glycoliposomes can be obtained by exploiting the self-assembling propensity of palmitoylated peptides (Pam_2_ and Pam_3_), which are known for their strong immunomodulating properties involving the activation of the heterodimeric receptors TLR1/TLR2 and TRL2/TLR6 [50]. For example, a synthetic oligosaccharide mimicking the O-antigen of Shigella flexneri 2a lipopolysaccharide and the influenza hemagglutinin peptide HA_307–319_, used as a T helper epitope, were respectively assembled into 70 to 90 nm vesicles and conjugated to the maleimide group of Pam_3_CAG [39]. This fully synthetic nanovesicles triggered specific antibody responses against the glycoantigen when administered intramuscularly to mice and provided protection against an experimental challenge with S. flexneri 2a [39]. The obtained immune response was associated with the dense exposition of the glycoantigens, resulting in the clustering and activation of B-cells, and the high cellular uptake primed the T helper cells [39]. Moreover, the Tn antigen was conjugated to a the T-cell epitope YAF, a 20-residue peptide derived from a protein of the outer membrane of N. meningitidis, and the glycopeptide was linked to Pam_3_Cys to form a 100 nm diameter liposomal vaccine formulation [40,51]. This liposomal formulation triggered a robust production of glyco-specific IgG, involving the maturation of dendritic cells and the activation of T helper cells by means of the built-in adjuvant Pam_3_Cys and the YAF T-cell epitope peptide [40].

In an alternative strategy, the Tn antigen was first conjugated to an unnatural amino acid, α-methylserine, and the glycosylated α-methylserine was incorporated into the most immunogenic region of the protein mucin 1 (MUC1), which is overexpressed on the surface of cancer cells [52,53]. A T helper epitope from human poliovirus 1 was also incorporated and these different moieties were conjugated to the TLR2/TLR6 agonist Pam_3_Cys. The incorporation of this glycolipopeptide into a phospholipid-based liposomal formulation resulted in the elicitation of a robust IgG antibody response against the MUC1-Tn antigen upon mice intradermal immunization, with the predominance of the IgG3 subclass, which is typical for carbohydrate-specific responses [41,54]. This work was initially inspired by Boons and colleagues who used a similar glycolipopeptide, but with the glycoantigen attached directly to Pam_3_CSK_4_ [42,55]. The liposomal formulation incorporating Pam_3_CSK_4_ and the QS-21 adjuvant, a mixture of saponin molecules from the Q. Saponaria tree, triggered high titers of antigen-specific IgG, with the predominance of IgG1, which has roles in the Th2 (humoral) response, and IgG2a, which participates in cellular cytotoxicity [42,56,57].

The glycolipid α-galactosylceramide (α-GC), a natural amphiphilic adjuvant molecule, has also been exploited for the design of self-assembled lipid nanocarriers. Synthetic α-GC can activate invariant natural killer T (iNKT) cells through binding to TCRs, which then triggers the release of immunostimulatory cytokines [58,59]. A liposomal nanovaccine combining the sialyl-Tn (sTn) antigen and the α-GC glycolipid as a built-in adjuvant proved to be very effective in stimulating the IgM-to-IgG switch of anti-sTn antibodies [43]. Similarly, the Tn glycoantigen was covalently attached through the carbamate moiety of α-GC and co-formulated within liposomes with a diameter between 100 and 400 nm to generate a self-adjuvated vaccine that produced robust anti-Tn IgG antibody responses and enhanced affinity maturation, a key process to generate antibodies with increased affinity, avidity and ability to neutralize invading pathogens [44]. Another liposomal vaccine formulation with the *S. pneumoniae* serotype 14 polysaccharide (Pn14PS) and a glycolipid derived from α-GC (PBS57), known as an iNKT cell activator, led to high levels of IgG against the *S. pneumoniae* antigen upon immunization in mice [45]. Notably, the IgG titers induced by this fully synthetic formulation were higher than those observed with the commercialized Prevnar13 vaccine, a 13-valent pneumococcal glycoconjugate vaccine using the protein CRM197 as an immunogenic carrier [45,60]. This study showed that not only is the α-GC critical for inducing a robust humoral response, but the liposomal nanostructure also plays an important role for the high production of anti-Pn14PS IgG [45].

The TLR4 agonist MPLA, an analog of lipid A of the outer membrane of Gram-negative bacteria, has also been harnessed for the design of lipid nanocarriers for synthetic saccharide antigens [61]. The activation of membrane-bound TLR4 of macrophages and dendritic cells by MPLA leads to the secretion of pro-inflammatory cytokines and chemokines, which create a microenvironment conducive to antigen presentation and the recruitment of immune cells [61,62,63]. Additionally, MPLA stimulates the production of antigen-specific antibodies by B-cells, contributing to long-lasting humoral immunity [63]. Owing to these immunostimulant properties, MPLA has been investigated as a potential carrier for polysaccharides. For instance, a tetrasaccharide derived from the extremity lipoarabinomannan (LAM) capsular polysaccharide of M. tuberculosis was conjugated to MPLA, which was then incorporated into liposomes composed of distearoylphosphatidylcholine and cholesterol, yielding a robust IgG antibody response against the carbohydrate component upon immunization in mice [46]. Additionally, α-2,9-polysialic acid from the capsular polysaccharide of N. meningitidis group C was conjugated to MPLA. A high secretion of anti-sialic acid IgG was measured upon immunization in mice, with mainly IgG2b and IgG2c isotypes, which are important for the T-independent response and the Th1 (cellular) response, respectively [46,47,56,64]. Furthermore, the antisera bound avidly to the capsular polysaccharide of N. meningitidis group C cells, showing that the antibodies can be directed to the bacteria [47]. MPLA was also investigated as a potential carrier molecule and natural enhancer for producing a fully synthetic glycoconjugate vaccine for cancer immunotherapy. This study revealed that liposomes incorporating MPLA conjugated to GM3, a glycoantigen expressed on melanoma and other cancer cells, produced a robust IgG antibody response in mice, with IgG3 as a main subclass [48]. These examples highlight the great potential of MPLA as a carrier for glycoantigen owing to its self-adjuvating properties.

### 3.2. Gold Nanoparticles

Inorganic nanoparticles, including gold nanoparticles (AuNPs) and silica nanoparticles, have gained attention for their potential use in vaccine development due to their physicochemical properties that improve antigen stability and targeting [65,66,67]. Inorganic nanoparticles can be easily functionalized, are usually biocompatible, and can be customized in terms of their shape and size [65]. These characteristics modulate their recognition and uptake by APCs, with larger spheric AuNPs being demonstrated to be more effective for antibody production [32]. For example, polyethylene glycol (PEG)-Tn complexes, with different densities of the glycoantigen, were conjugated to 5 to 20 nm spherical AuNPs (Table 2). The resulting nanoparticle-based glycoconjugate vaccine elicited a robust antigen-specific immune response, as evidenced by the ability of the resulting antibodies to recognize naturally occurring Tn-antigen glycans and mucins found on mammalian cells [68]. Notably, the density of the Tn antigen, which plays an important role in B-cell activation, conjugated on the PEG chain had a significant impact on the immune response. In fact, the highest IgG titers were reported for glyco-AuNPs with PEG_25_Tn_25_ and PEG_80_Tn_20_ [68]. Additionally, three to five nm spherical AuNPs coated with a peptide derived from the C3d protein used as a B-cell adjuvant and a peptide derived from tumor-associated mucin 4 (MUC4) and conjugated with Thomsen–Friedenreich (TF; Gal-GalNAc) were found to elicit anti-TF IgM and IgG antibodies upon immunization in mice [69].

AuNPs have also been investigated for the development of bacterial glycoconjugate vaccines. In a recent study, a synthetic tetrasaccharide epitope related to the *S. pneumoniae* serotype 14 polysaccharide (Pn14PS) was conjugated to two nm spherical AuNPs in combination with a T helper peptide (OVA_323–339_), and this formulation was injected subcutaneously in mice [70]. This resulted in the robust production of specific anti-Pn14PS IgG antibodies. Moreover, splenocytes isolated from these immunized mice showed increased TNF-α, IL-2 and IL-5 secretion upon stimulation with OVA_323–339_, indicating the activation of memory T-cells [70]. Similarly, fragments of serotype 14 (Pn14PS) and 19F (Pn19F) of *S. pneumoniae* and a T helper peptide (OVA_323–339_) were linked to AuNPs [71]. Interestingly, when mice were immunized with these two nm AuNPs, the presence of both Pn19F and Pn14PS on the same AuNPs significantly increased specific IgG antibody levels against Pn14PS compared to nanoparticles displaying Pn14PS alone. In sharp contrast, no anti-Pn19F IgG was generated, suggesting that the use of a longer polysaccharide fragment with multiple repeating units may be necessary to create a conformational epitope that can effectively activate the immune system [71]. In another study, the terminal-branched hexaarabinofuranoside fragment (Ara6) from lipoarabinomannan and arabinogalactan were conjugated to AuNPs and evaluated in rabbits [72]. These polysaccharides constitute the primary component of the cell wall of *Mycobacterium tuberculosis*, the etiologic agent of tuberculosis [73]. The antisera of immunized rabbits showed high levels of anti-Ara6 specific antibodies that bind to the surface of *Mycobacteria* cells [72]. Overall, these studies highlight that AuNPs demonstrate significant potential as a delivery nanoplatform for glycoantigens for vaccines to prevent bacterial infections and fight cancers.

### 3.3. Virus-like Particles

Virus-like particles (VLPs) are composed of self-assembling proteins usually originating from viral capsid proteins that mimic the structure of viruses, but lack genetic materials, making them safe vaccines [74]. VLPs can display multiple copies of the antigen in a repetitive manner on their surface, promoting the recognition, uptake and activation of APCs. These proteinaceous nanoparticles have been successful in eliciting strong immune responses against the grafted antigen and are currently used in human vaccines, as exemplified with the licensed VLP-based vaccines against human papillomavirus (HPV), hepatitis B and E, malaria and coronavirus [74,75].

Bacteriophage Qβ is a single-stranded RNA virus known to infect Gram-negative bacteria by their pili and has been used for the design of VLPs for antigen delivery, including glycoantigens [76,77]. In a study, MUC1-TF and MUC1-sTn glycopeptides were linked to bacteriophage Qβ VLPs by amide coupling, and the immunogenicity of these nanoparticles was evaluated in transgenic mice expressing human MUC1 (Table 3) [78]. The sera IgG antibodies generated in response to the Qβ-MUC1-TF construct exhibited a stronger binding affinity to the B16-MUC1 melanoma cells, compared to the IgG antibodies induced upon immunization with the soluble forms of unglycosylated MUC1 peptide or MUC1-sTn, highlighting the key contribution of the VLP nanocarrier. Furthermore, vaccination with Qβ-MUC1-TF provided significant protection against cancer cells in a model of tumor metastasis [78].

Another study demonstrated that presenting the Tn glycoantigen on bacteriophage Qβ VLP triggers strong humoral immune responses against the glycoantigen. The impact of different adjuvants, exposition of the antigens and vaccine dosage on the intensity and the isotype antibodies were also investigated. It was observed that the local concentration of antigens at the injection site, rather than the overall dose administered, was an important factor for generating high Tn-specific IgG levels [79]. The IgG antibodies generated showed high levels of specificity and affinity towards endogenous Tn antigens located on the surface of human leukemia cells, supporting the potential of the Qβ-Tn nanovaccine to fight cancers [79]. The conjugate MUC1-β-TF with bacteriophage Qβ carrier triggered the production of high levels of IgG that could recognize diverse glycoforms of the tumor-associated MUC1 antigen. These antibodies were also able to target and eliminate tumor cells [80]. The bacteriophage Qβ nanocarrier was also evaluated for the delivery of bacterial polysaccharides. Synthetic fragments of *S. pneumoniae* serotype 3 and 14 capsular polysaccharides were conjugated onto bacteriophage Qβ VLPs by copper-mediated azide-alkyne cycloaddition, which was possible because of the addition of an N-hydroxysuccinimide-alkyne linker on the primary amine of the carrier. These glycoconjugates stimulated specific IgG against both glycoantigens and protected mice against *S. pneumoniae* infections [81]. As a proof of concept, the capsular polysaccharide of serotype II (PSII) *Streptrococcus agalactiae* was conjugated Qβ, and it was observed that a single dose of this glycoconjugate nanovaccine elicits the robust production of IgG and opsonic antibodies against the PSII [82].

The capsid from the cowpea mosaic virus (CPMV) was also used as a carrier for synthetic saccharide antigens in the context of cancer immunotherapy. The Tn antigen was conjugated on the CPMV capsid by means of an orthogonal conjugation between a maleimide group on the sugar unit and two non-native cysteine residues on the VLP [83]. When injected to mice subcutaneously, this formulation triggered a strong immune response, with sera IgG antibodies effectively interacting with Tn antigens on the surface of breast cancer cells [83]. These studies indicate that VLPs, which have been proven to be efficient for the delivery of proteinaceous antigens, can also be exploited for the delivery of synthetic glycoantigens.

### 3.4. Dendrimers

Dendrimers are highly branched polymers with defined three-dimensional architectures and sizes that lead to distinctive chemical and biological properties [84]. Their surface can be easily functionalized, facilitating the orthogonal conjugation of various molecules, including oligosaccharides [84]. Along with their capacity to stimulate the immune system through their surface multivalency, the flexibility of dendrimers enables the generation of precise nanostructures with tailored characteristics, making nanomaterials appealing for vaccination [84,85]. For example, a cluster of three Tn antigens containing a T-cell epitope from the poliovirus (PV) was linked to a lysine scaffold to generate a highly branched dendrimer (Table 4). Upon immunization in mice, high levels of anti-Tn IgG that recognize cancer cells were produced and provided protection in a tumor challenge study [86]. Subsequently, Lo-Man and colleagues investigated the effects of using different residues for presenting the Tn glycoantigen, such as serine (Ser), threonine (Thr) and homoserine (hSer) [87]. While the antibodies generated with the unnatural residue Tn-hSer did not recognized tumor cell lines, the ones from mice immunized with the Tn-Ser and Tn-Thr led to anti-Tn antibodies that avidly bind tumor cell lines [87]. Subsequently, the T-cell epitope from PV was replaced by epitopes derived from tetanus toxoid (TT_830–844_) and the Pan DR ‘universal’ T helper epitope (PADRE) for a more humanized formulation. Both vaccine formulations elicited high titers of Tn-specific antibodies [87]. The antibodies from the TT(_830–844_)-functionalized dendrimers recognized and promoted the killing of tumor cell lines [87]. Finally, the TT_830–844_ formulation was prepared under GMP conditions and evaluated in a phase I clinical trial for its potential usage as a breast cancer therapeutic vaccine [88]. The results demonstrated that this fully synthetic vaccine formulation elicited the production of anti-Tn IgG in all immunized individuals without any significant side effects. Furthermore, these antibodies were able to recognize cancer cell lines presenting the Tn glycoantigen and to eliminate cancer cells by the mobilization and activation of the complement system [89]. To the best of our knowledge, no additional clinical trials were conducted with this formulation.

## 4. Chemical Strategies for the Conjugation of Synthetic Glycoantigens to Nanocarriers

The covalent attachment of antigenic polysaccharides purified from pathogens to carrier proteins has been historically achieved through reductive amination between the amine groups on the exposed Lys side chains and the carbonyl groups of the carbohydrates [48,90]. Alternatively, polysaccharide activation with 1-cyano-4-dimethylaminopyridine tetrafluoroborate (CDAP) and subsequent conjugation onto the Lys amine groups have also been exploited [91]. For example, the approved pneumococcal vaccines PCV7 (Pfizer) and Pneumosil (Serum Institute of India) are obtained from the conjugation of *S. pneumoniae* CPS to CRM_197_ through reductive amination [42,90,92] and CDAP chemistry [93], respectively. While robust, these approaches can prove inefficient and do not allow for the precise loading and orientation of the antigens [94]. The low selectivity of reductive amination can also result in undesired molecular cross-linking. Moreover, the isolation of polysaccharides from bacterial pathogens does not result in homogenous glycoconjugates and often requires a further chemical transformation for covalent linkage to immunogenic carriers. The need for higher levels of versatility and control over glycan structure, antigen density, purity and homogeneity has encouraged the use of synthetic glycans [48,90]. Additionally, de novo synthesis eliminates the risks associated with pathogen contamination caused by improper purification when using biological material [95].

### 4.1. Preparation of Synthetic Glycoantigens

Synthetic glycans offer key advantages over polysaccharides isolated from living organisms for the preparation of glycoconjugate nanovaccines, including stereochemical control and customized functionalization [96]. Synthetic glycans can display a diversity of unnatural functionalities intended for the ligation of the nanocarriers, including alkyne, aldehyde and azido groups [97]. Nonetheless, the synthesis of oligosaccharides remains complex and challenging. Glycans are usually obtained through linear multistep and/or convergent synthesis [96], whereas alternative strategies, such as one-pot synthesis, solid-phase synthesis and enzyme-assisted synthesis, have shown great potential [98]. For instance, two analogs of the tumor-associated Lewis Y (Le^y^) glycoantigen were synthesized through multiple cycles of orthogonal deprotection and glycosylation in a single reaction vessel [99]. This automated method exploited the variations in anomeric center reactivity amongst protected thioglycoside building blocks and harnessed the activation of their thiols [96]. These synthetic glycans were then modified with disuccinimidal glutarate to obtain active esters for coupling to the primary amines of the protein KLH [99]. By exploiting the regiospecificity and stereoselectivity of glycosyltransferases and glycosidases, enzymatic biosynthesis can be combined with chemical synthesis to obtain pure and homogenous glycoantigens with a high yield [100,101]. Notably, enzyme-assisted synthesis can convert monosaccharides into specific oligosaccharides, without the use of protecting groups [102,103]. For example, a homogenous polysaccharide derived from *Neisseria meningitidis* serogroup W (NmW) CPS was prepared using a sequential one-pot multienzyme (OPME) reaction [100,101]. Starting with commercially available *N-*acetylneuraminic acid (Neu5Ac) and galactose as building blocks, size-controlled semi-synthetic NmW oligosaccharides were assembled enzymatically [100].

Notably, glycans are often attached to the hydroxyl group of the side chain of Ser and Thr residues to mimic *O*-glycosylation, which is an important protein post-translational modification that participates in viral infection and that can be used to differentiate cancer cells from healthy cells by the immune system [104,105,106]. Usually, the glycans are first attached to the side chain before the subsequent insertion of the building block into the peptide chain assembled by solid-phase peptide synthesis (SPPS) [86]. Through repeated coupling and deprotection cycles, SPPS for allows the chemical synthesis of a diversity of glycopeptides that can be orthogonally conjugated to diverse nanocarriers upon cleavage from the solid support [107]. For SPPS, the reactive groups of the glycosylated amino acid are modified with orthogonal protecting groups, including acetyl (glycan) and *Fmoc* (N-terminus amine), providing the free carboxylic acid for coupling with the deprotected amine group of the elongated peptidyl-resin [95,108,109]. For example, a protected methylserine functionalized with the Tn antigen was incorporated into an 11-residue peptide derived from the MUC1 antigen through standard SPPS [104,105]. While being an efficient and versatile approach, SPPS requires the careful selection of the solid support, linker, protective groups and cleavage strategy [108,110]. Notably, the hydroxyl groups of the glycan usually need to be protected by acetylation to avoid side reactions, requiring deacetylation after cleavage from the solid support and adding additional steps to the synthesis scheme [95,108,109]. Moreover, the potential hydrolysis of the glycosidic linkages under the harsh acidic conditions of the cleavage of the peptide from the resin needs to be considered when elaborating a synthesis scheme for glycopeptides [109].

Overall, these approaches have allowed for the synthesis of diverse glycoantigens, either as oligosaccharides or glycopeptides, that adequately mimic the naturally occurring glycans on the cellular target, which can then be easily attached to the nanocarrier of choice. The chemical synthesis of glycans and glycoconjugates is a complex and vast subject that is beyond the scope of the present review, and several excellent and highly comprehensive reviews have been published over the years [6,98,111,112,113].

### 4.2. Conjugation of Glycans and Glycopeptides to Nanocarriers

**Figure 2:**
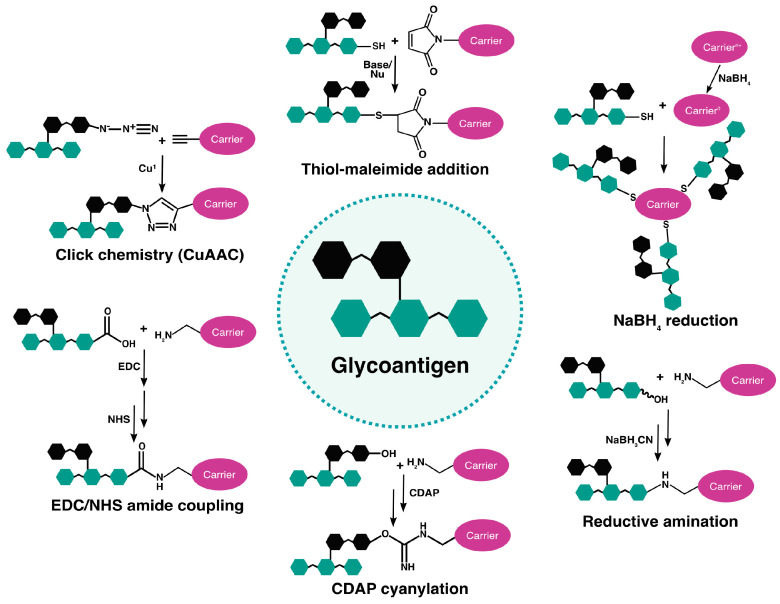
Overview of the strategies for glycan antigens’ chemical conjugation to nanocarriers. EDC: 1-ethyl-3(3-dimethylaminopropyl)carbodiimide; NHS: sulfo-N-hydroxysuccinimide.

As for polysaccharides isolated from bacteria, synthetic glycans can be attached to nanocarriers through reductive amination. This reaction takes place when a carbonyl group from the saccharide unit reacts with a primary amine on the carrier to form an imine (Schiff base), which is subsequently reduced to a secondary amine by reduction, often using NaBH_3_CN or NaBH_4_ (Figure 2) [114]. This process can sometimes be preceded by periodate oxidation to increase the number of available aldehyde groups on the glycans [11]. A reduction reaction can also take place between a synthetic glycan, or glycopeptide, and an inorganic nanocarrier, such as AuNPs, to form multivalent bonds. For example, the MUC4 glycopeptide antigen containing a thiol group was conjugated to gold through NaBH_4_ reduction, and the resulting glyco-AuNPs triggered a robust anti-glycan humoral response [69].

Synthetic glycoantigens can be linked to nanocarriers by means of the formation of an amide bond. This conjugation approach is often based on the use of 1-ethyl-3(3-dimethylaminopropyl)carbodiimide (EDC) as a crosslinking molecule able to form amide bonds between a carboxylic acid and a primary amine without inserting itself into the final product (Figure 2) [115]. EDC reacts with a carboxylic group and forms an unstable o-acylisourea ester intermediate that promptly reacts with a primary amine, leading to the formation of the amide bond and the release of an isourea by-product [115]. The introduction of sulfo-*N*-hydroxysuccinimide (NHS) stabilizes the reactive o-acylisourea intermediate through the formation of an NHS-ester, allowing for a higher reaction yield [115,116]. This strategy based on EDC chemistry was used for the synthesis of monovalent and tetravalent Tn-calixarene glycoconjugates, which induced robust anti-Tn IgG production in immunized mice [117].

Copper-mediated azide-alkyne cycloaddition (CuAAC), also known as “click chemistry”, has proven to be an efficient tool for the selective coupling of glycoantigens and glycopeptides to nanocarriers [102,103]. Using copper as a catalyst, this reaction involves the stereospecific formation of a stable triazole ring between the azide of one coupling partner and the alkyne moiety of the other (Figure 2) [102]. Notably, the lack of the azide group from virtually all natural compounds and its low reactivity in aqueous solution confer a high level of bio-orthogonality to this reaction [102,118,119]. For example, an azide-functionalized derivative of the tumor-associated carbohydrate TACA antigen GM3 was coupled to MPLA containing a propargyl group, resulting in a liposomal glycoconjugate nanovaccine [48]. As copper toxicity constitutes a notable concern, copper-free conditions and catalysts have been developed for biomedical applications, although lower yields and/or reaction rates are often observed [120]. Notably, the presence of a triazole link between the TA3Ha tumor-associated carbohydrate antigen and the Qβ bacteriophage VLP, used as the nanocarrier, significantly reduced the level of anti-TA3Ha antibodies in immunized mice, with some antibodies directed towards the triazole linker [121]. This observation emphasizes that the choice of linker between the saccharidic antigen and the nanocarrier is critical to achieve the desired immunogenicity of glycoconjugate nanovaccines.

The orthogonal maleimide-thiol Michael addition that leads to the formation of a stable thioether bond has also been employed for the preparation of glycoconjugates. For instance, a synthetic sulfhydryl derivative of the *Shigella flexneri* O-specific polysaccharide antigen was linked to a maleimide-functionalized lipopeptide (Pam_3_CAG) to generate self-adjuvanted glycoliposomes [39]. Due to the lower prevalence of thiols on proteins compared to primary amines, the maleimide-thiol addition has a higher specificity than reductive amination or EDC/NHS chemistry [122]. Moreover, since thiol groups can be generated either through the reduction of intrinsic disulfide bonds or through common addition reactions with primary amines, this reaction can be performed with a wide variety of nanocarriers [123,124]. Other coupling strategies have also been explored for the formation of covalent bonds between synthetic glycoantigens and nanocarriers. For example, a sialyl-Tn antigen displaying an azide moiety was linked to an α-galactosyl-ceramide (αGalCer) immunostimulating molecule through the hydrogenolysis of the azide and subsequent amidation of the amine with *p*-nitrophenylester [43].

Overall, the covalent attachment of synthetic glycans to nanocarriers can be realized through a diversity of ligation strategies, each with their own advantages and limitations (Table 5). A thorough examination of the physicochemical properties of the antigen, linker and nanocarrier, the reactive conditions, as well as the production time and costs is critical to elaborate the optimal conjugation strategy. Notably, it is known that the linker position, length and chemical structure affect the orientation of the glycoantigen grafted on the surface. In turn, this modulates the glycan’s presentation to immune receptors and influence the resulting immunogenicity of the glycoconjugates, as well as the binding affinity and specificity of the generated antibodies [125]. These observations further highlight the importance of carefully selecting the conjugation chemistry for the development of glycovaccines.

## 5. Conclusions

Glycans are highly prevalent on the cell surface of all living organisms, and they constitute key molecular targets for the development of subunit vaccines [6,7]. However, the use of soluble polysaccharides as vaccines leads to short-lived immune responses, as it induces T-cell-independent immunity [7,8,22]. Consequently, this approach fails to activate T-cells, a critical step for generating long-lasting immunity [23]. To address this limitation, saccharide antigens are usually attached to immunogenic protein carriers, albeit this approach complexifies production and can lead to undesirable immunogenic interference [12]. Recently, nanoparticle-based delivery systems have shown great potential to overcome these limitations [8,13]. Different types of nanoparticles, such as liposomes assembled from lipidic TLR agonists, gold nanoparticles, VLPs and dendrimers, have been explored for glycoconjugate vaccines. The versatility of nanoparticles and their ability to induce robust immune responses makes them attractive delivery platforms for vaccines and immunotherapies targeting glycoantigens. As research in this field continues to progress, we can expect further developments and innovative applications of nanoparticle-based glycoantigen delivery systems that could significantly improve the prevention of infectious diseases and the treatment of cancers. Notwithstanding, there are still some challenges to address in order to elicit the suitable immune response against glycoantigens, which will require a better understanding of the influence of the conjugation chemistry and of the chemical nature of the nanocarriers in the immune responses against the grafted glycans [25]. The conjugation chemistry and the linker both play a critical role in the presentation of the glycoantigen to immune receptors, which modulates the immunogenicity of the glycoconjugate. In some cases, the antibodies generated could be less specific and/or have a lower binding affinity toward the naturally occurring glycoantigens, requiring careful design. As a diversity of glycans are naturally present on human cells and the bacteria constituting our microbiome [128], synthetic glycoconjugate vaccines need to be carefully designed to address potential molecular mimicry between the pathogenic glycoantigen and the host’s glycans. Progress in fundamental glycobiology and in our understanding of how oligosaccharides interact with the immune system will further pave the way to more effective, safer and affordable glycoconjugate vaccines.

## Figures and Tables

**Table 1:** Liposomal carriers for the delivery of bacterial and cancer glycoantigens.

Nanocarrier	Antigen	Conjugation Chemistry	Additional Adjuvant	Immune Responses	Ref.
**Cholesterol**	Tn	Copper-mediated azide-alkyne cycloaddition (CuAAC)	CpG	Anti-Tn IgG production IFN-γ secretion	[38]
**Pam_3_CAG**	Three repeating units of O-antigen from *S. flexneri* and Influenza peptide (HA_307–319_)	Thiol-maleimide	None	Specific antibodies and protection against *S. flexneri*	[39]
**Pam_3_Cys**	Tn-YAF (T helper)	C-term amide coupling	None or QS21	Anti-Tn IgG production	[40]
**Pam_3_CysSK_4_**	T helper- MUC1-Tn	Amide coupling on solid support	None	Anti-MUC1-Tn IgG Predominant IgG3	[41]
**Pam_2_CysSK_4_ and Pam_3_CysSK_4_**	T helper- MUC1-Tn	Amide coupling on solid support	None or QS-21	Anti-MUC1-Tn IgG higher with Pam_3_ Predominant IgG1 and IgG2a	[42]
**α-galactosylceramide**	sTn	Amide coupling via diselenoester	None	Anti-sTn IgG production	[43]
	Tn	Amide coupling	None	Anti-Tn IgG production Affinity maturation	[44]
**PBS150**	Tetrasaccharide of *S. pneumoniae* serotype 14	Amide coupling	None	Specific IgG against *S. pneumoniae* 14	[45]
**Monophosphoryl lipid A (MPLA)**	Lipoarabinomannan of *M. tuberculosis*	Copper-mediated azide-alkyne cycloaddition (CuAAC)	None	Specific IgG against *M. tuberculosis* Predominant IgG1	[46]
	α-2,9-Polysialic acid capsular polysaccharide of *N. meningitidis* group C	Amide coupling	None, Alumn, CFA or TiterMax Gold	Specific IgG against *N. meningitis* group C Predominantly IgG2b and IgG2c	[47]
	GM3	Amide coupling or Copper-mediated azide-alkyne cycloaddition (CuAAC)	None or TiterMax Gold	Anti-GM3 IgG production Predominant IgG3	[48]

CpG: unmethylated 5′-C-phosphate-G-3′; HA: hemagglutinin; Tn: N-acetylgalactosamine Thomsen nouveau; sTn: sialyl-Tn; CFA: complete Freund’s adjuvant.

**Table 2:** Gold nanoparticles as carriers for the delivery of glycoantigens.

Nanocarrier	Antigen	Conjugation Chemistry	Additional Adjuvant	Immune Responses	Ref.
**AuNP**	Tn	NaBH_4_ reduction	None	Anti-Tn IgG production Recognition of mucins presenting different form of Tn	[68]
	TF-MUC4 and C3d peptide	NaBH_4_ reduction	None	Anti-MUC4-TF IgM and IgG production	[69]
	Oligosaccharide of *S. pneumoniae* serotype 14 and OVA_323–339_	Oxidation reduction (S-Au)	MPLA and Quil-A	Anti-Pn14PS IgG production TNF-α IL-4 and IL-5 production	[70]
	Oligosaccharide of *S. pneumoniae* serotype 14 and 19F, and Ova_323–339_	Oxidation reduction (S-Au)	Quil-A	Anti-Pn14PS IgG production	[71]
	Hexaarabinofuranoside fragment (Ara6) of lipoarabinomannan of *M. tuberculosis*	Oxidation reduction (S-Au)	Complete Freund	Specific antibodies against *Mycobacteria* cells	[72]

Legend: Tn: Thomsen nouveau; TF: Thomsen–Friedenreich; MUC4: mucin 4 peptide; OVA: ovalbumin; MPLA: monophosphoryl-Lipid A.

**Table 3:** VLPs as nanocarriers for the delivery of synthetic glycoantigens.

Nanocarrier	Antigen	Conjugation Chemistry	Additional Adjuvant	Immune Responses	Ref.
**Bacteriophage Qβ**	MUC1-TF and MUC1-sTn	Amide coupling	MPLA	Anti-MUC1-TF and anti-MUC1-sTn IgG production Protection against cancer cells	[78]
	Tn	Copper-mediated azide-alkyne cycloaddition (CuAAC)	Complete Freund or TiterMax Gold or Alum	Anti-Tn IgG production and strong binding with human leukemia cells	[79]
	MUC1-β-TF	Amide coupling	MPLA	Anti-MUC1-β-TF IgG production which can eliminate tumor cells	[80]
	Tetrasaccharide of *S. pneumoniae* serotype 3 et 14	Copper-mediated azide-alkyne cycloaddition (CuAAC)	αGC	Specific IgG against *S. pneumoniae* 3 and 14	[81]
	Capsular polysaccharide of *S. agalactiae* 2	Reductive amination	Alumn	Specific IgG against *S. agalactiae* 2	[82]
**Cowpea Mosaic Virus** **(CPMV)**	Tn	Thiol-maleimide	Complete Freund	Anti-Tn IgG production and strong binding with breast cancer cells	[83]

Legend: MUC1, mucin 1 peptide; TF, Thomsen–Friedenreich; sTn, sialyl-Tn; MPLA, monophosphoryl lipid A; Tn, Thomsen nouveau; αGC, α-galactosylceramide.

**Table 4:** Dendrimeric carriers for the delivery of cancer glycoantigens.

Nanocarrier	Antigen	Conjugation Chemistry	Additional Adjuvant	Immune Responses	Ref.
**Tetravalent lysine core**	3Tn-PV	Amide coupling on solid support (Pfp-ester)	Alumn	Anti-Tn IgG production Protection against tumor	[86]
	3Tn(S or T or hS)-PV	Amide coupling on solid support (Pfp-ester)	Alumn	Anti-Tn IgG production and recognition of tumor cells	[87]
	3Tn-PADRE or 3Tn- TT_830–844_	Amide coupling on solid support (Pfp-ester)	Alumn and CpG	Anti-Tn IgG production and recognition and killing of tumor cells	[87]
	3Tn-TT_830–844_	Amide coupling on solid support (Pfp-ester)	AS-15	Anti-Tn IgG production and recognition and killing of tumor cells IFN-y production	[88]

Legend: Tn: Thomsen nouveau; PV: polio virus peptide; Pfp: pentafluorophenyl; PADRE: Pan DR ‘universal’ T helper epitope; TT: tetanus toxoid peptide; CpG: unmethylated 5′-C-phosphate-G-3′.

**Table 5:** Chemical strategies for conjugation of glycans on nanocarriers.

Conjugation Strategy	Advantages	Limitations	Ref.
**Copper-mediated azide-alkyne cycloaddition**	Orthogonal reaction Mild and simple reactive conditions High yield Stereospecific Vast array of conjugation partners Minimal, or no purification needed	Requires non-native azide and alkyne groups Copper catalyst can be cytotoxic if not properly removed Triazole linker can lead to low immunogenicity Antibodies can target the triazole linker	[102,103,118,120,121]
**Thiol-maleimide addition**	Orthogonal reaction High yield Mild reaction conditions	Side reactions can occur (thiazine formation) Requires available thiol group and maleimide functionalization	[123,126,127]
**Reductive amination**	Ubiquitous carbonyl groups on glycans Useful for conjugation to peptide, protein and inorganic carriers Variety of effective reducing agents	Low specificity Heterogenicity of the final product Undesirable cross-linkage	[69,70,71]
**EDC/NHS ligation**	High yield and reaction rate Carboxylic groups can be easily added to glycans Short linker (amide bond) Requires primary amine group on the carrier	Optimization may be required Protection of non-targeted carboxylic acids needed	[41,115,116,117]

Legend: EDC: 1-ethyl-3(3-dimethylaminopropyl)carbodiimide; NHS: sulfo-N-hydroxysuccinimide.

## Data Availability

No new data reported.
